# Machine Learning Neuroprotective Strategy Reveals a Unique Set of Parkinson Therapeutic Nicotine Analogs

**Published:** 2020-03-20

**Authors:** Felipe Rojas-Rodríguez, Carlos Morantes, Andrés Pinzón, George E. Barreto, Ricardo Cabezas, Leonardo Mariño-Ramírez, Janneth González

**Affiliations:** 1 Departamento de Nutrición y Bioquímica, Pontificia Universidad Javeriana. Bogotá D.C, Republic of Colombia; 2 Departamento de Biología, Universidad Nacional de Colombia. Bogotá, Republic of Colombia; 3 Instituto de Genética, Universidad Nacional de Colombia, Bogotá, Republic of Colombia; 4 Department of Biological Sciences, University of Limerick, Limerick, Ireland; 5 National Center for Biotechnology Information, National Library of Medicine, National Institute of Health, 8600 Rockville Pike, Bethesda, MD 20894, USA

**Keywords:** Parkinson’s disease, Nicotine analogs, PI3K/AKT, Machine learning, Artificial Neural Networks, Substanita Nigra

## Abstract

**Aims:**

Present a novel machine learning computational strategy to predict the neuroprotection potential of nicotine analogs acting over the behavior of unpaired signaling pathways in Parkinson’s disease.

**Background:**

Dopaminergic replacement has been used for Parkinson’s Disease (PD) treatment with positive effects on motor symptomatology but low progression and prevention effects. Epidemiological studies have shown that nicotine consumption decreases PD prevalence through neuroprotective mechanisms activation associated with the overstimulation of signaling pathways (SP) such as PI3K/AKT through nicotinic acetylcholine receptors (*e.g* α7 nAChRs) and over-expression of anti-apoptotic genes such as Bcl-2. Nicotine analogs with similar neuroprotective activity but decreased secondary effects remain as a promissory field.

**Objective:**

The objective of this study is to develop an interdisciplinary computational strategy predicting the neuroprotective activity of a series of 8 novel nicotine analogs over Parkinson’s disease.

**Methods:**

We present a computational strategy integrating structural bioinformatics, SP manual reconstruction, and deep learning to predict the potential neuroprotective activity of 8 novel nicotine analogs over the behavior of PI3K/AKT. We performed a protein-ligand analysis between nicotine analogs and α7 nAChRs receptor using geometrical conformers, physicochemical characterization of the analogs and developed manually curated neuroprotective datasets to analyze their potential activity. Additionally, we developed a predictive machine-learning model for neuroprotection in PD through the integration of Markov Chain Monte-Carlo transition matrix for the 2 SP with synthetic training datasets of the physicochemical properties and structural dataset.

**Results:**

Our model was able to predict the potential neuroprotective activity of seven new nicotine analogs based on the binomial Bcl-2 response regulated by the activation of PI3K/AKT.

**Conclusion:**

Hereby, we present a robust novel strategy to assess the neuroprotective potential of biomolecules based on SP architecture. Our theoretical strategy can be further applied to the study of new treatments related to SP deregulation and may ultimately offer new opportunities for therapeutic interventions in neurodegenerative diseases.

## 1. INTRODUCTION

Parkinson’s Disease (PD) is the second most common neurodegenerative disorder, mainly affecting the population over 60 years of age [1]. As a complex multifactorial disease, PD is caused by the accumulation of unfolded proteins, genetic predisposition, mitochondrial dystrophies, epigenetic imbalance and environmental factors that increase the degeneration of dopaminergic neurons in the *substantia nigra* of the brain [2 – 7]. Traditional approaches of dopaminergic replacement, such as levodopa, carbidopa, and dopamine agonists (apomorphine, amantadine, cabergoline, pergolide, etc) restore direct or indirect dopaminergic supply, modulate dopaminergic neuron activity and stimulate postsynaptic receptors [2, 8]. However, classical therapeutics are directed towards treating PD symptoms (dyskinesia, motor fluctuations and systemic complications) in exchange for reducing disease progression coupled with a lack of long-term efficacy [1, 2, 9].

Epidemiological studies demonstrated an inverse association between cigarette smoking and PD risk [10 – 12]. Pre-clinical studies using *in vivo* and *in vitro* models showed that nAChR agonists protect nigrostriatal and other neuronal cell populations against cytotoxic damage, suggesting a neuroprotective activity [12 – 15]. nAChRs are ligand-gated ion channels capable to respond to several ligands such as acetylcholine and nicotine [16]. Classified between neuronal and muscle subtype receptors, only one pentameric subtype of nAChR (2*α*1, *β*1, *γ*/*ε*, *δ*) has been reported for muscles compared with the 12 subtypes in the neural system (*α*2–10, *β*2–4). Neuroprotective activity of nicotine and nicotine agonists has been linked to the activation of α7-nAChRs [14, 17]. Highly expressed in dopaminergic neurons, α7-nAChRs activation increases calcium flow and activates downstream SP such as ERK/MAPK, JAK2/STAT3, calmodulin and PI3K/AKT. SP activation has been related with neuronal survival, synaptic plasticity and decreased apoptosis due to the overexpression of cell survival proteins such as Bcl-x, CREB and Bcl-2 [17 – 19]. In parallel, tumor suppressor p53 directly regulates cell survival by modulating Bax:Bcl-2 expression, influencing the apoptotic fate of a cell in response to stress [20]. Bcl-2 has been suggested as a candidate for the nicotine-mediated neuroprotection activity in neurons. For example, nicotine induces the phosphorylation of Bcl-2 through either protein kinase C (PKC) and the MAPKs ERK1 and ERK2 or through PI3K-induced phosphorylation of AKT causing the inhibition of apoptosis in neuronal cultures [14, 17, 18, 21 – 25]. Additionally, overexpression of Bcl-2 Prevents neuronal death in *Mus musculus* models and Bcl-2 inhibition results in viability loss in dopaminergic cells through the induction of caspase-3 [26, 27].

## 2. METHODS

**Fig. (1).**
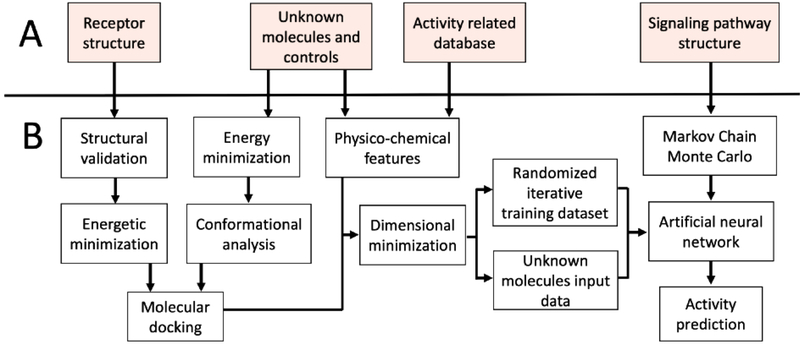
Diagrammatic workflow of the computational strategy for ligand prediction over SP architecture. The pipeline for the strategy implementation uses a receptor crystallographic structure, structure of the unknown ligands, known ligands data interacting with the receptor of interest and SP architecture. The SP must interact with the receptor by signaling cascade of secondary messengers and be triggered by the interactions of known and unknown ligands with the receptor. **A**) Input of biological information **B**) Computational methods to predict the activity of a series of ligands. Subgroups of computational methods aim to represent biological stages of the SP response to external ligands: 1) Structural validation, energy minimization, conformational search and molecular docking stand as protein ligand complex and 2) Markov Chain Monte Carlo and Artificial neural network as methods to generate a quantitative model with SP architecture embedded. Physico-chemical featuring, dimensional minimization and training/testing datasets were implemented to integrate the structural data with the systemic model of the SP and the machine learning model.

In this sense, nAChRs agonists can reduce secondary effects of classical therapies such as levodopa-induced dyskinesia, increased dopamine release and improve cytochrome P450 activity [28, 29]. Nicotine usage as a pharmacological agent is limited considering toxicity and addiction [30]. Nicotine analogs retaining neuroprotective activity, but reducing toxicity and addiction offer an excellent pharmacological alternative in PD [31 – 35]. We have previously the antioxidant potential of two nicotine analogs against rotenone-induced ROS generation in a PD *in vitro* model founding that 10 μM of (E)-Nicotinaldehyde O-Cinnamyloxime was able to reduce superoxide anion and hydrogen peroxide production in neuronal SH-SY5Y cells treated with rotenone for 24h [10]. Consistently, other studies showed that nicotine analogs decrease superoxide anion generation and oxidative stress in rodents *Mus musculus* and *Rattus rattus*, as well as primates *Macaca fascicularis* and *Saimiri sciureus* through the activation of α7 nAChRs and PI3K/AKT [17, 35 – 38]. Computational methods have been used to infer the activity of candidate molecules but, due to the intrinsic PD complexity, is important to improve drug scanning efficiency by integrating structural and systemic data [39 – 40]. The development of holistic computational methodologies for SP and their modulatory mechanisms can lead to the discovery of new pharmacological agents [40, 41]. In this aspect, the use of Artificial neural networks (ANN) have been previously used in a simplified model of the Toll-like receptor signaling pathway, and the PI3K/AKT pathway in the context of cancer [42]. Nevertheless, the absence of integrative modeling in PD has led to a decrease in drug discovery efficiency. In the present study, we predicted the effect of 8 novel nicotine analogs as possible neuroprotective agents acting on PI3K/AKT by integrating structural bioinformatic methods, SP manual reconstruction and ANN (Fig. 1).

## 3. RESULTS

### 3.1. Geometry and Structure Analysis

Structures of (3R,5S)-1, methyl-5-(piridine-3-yl) pirrolidine-3-ol (A1), 3-(1,3-dimethyl-4,5-dihidro-1h-pirazole-5-yl) piridine (A2), 3-(3-methyl-4,5-dihidro-1h-pira-zole-5-yl) piridine (A3), 3(((2S-4R)-1,4-dimethylpirrolidine-2-yl)) (A4), 3-((2S,4R)-4-(fluoromethyl)-1-methylpirrolidine-2-il)piridine (A5), 3-((2S,4R)-4-methoxi-1-methylpirrolidine-2-yl) piridine (A6), 3-((2S,3S)-1,3-dimethylpirrolidine-2-yl) piridine (A7) and 5-methyl-3-(piridine-3-yl)-4,5-dihidroisoxazole (A8) were used for subsequent methods (Table 1). The neutral molecules optimized at the B3LYP/631G level and conformationally analyzed at the PM6 level showed rotations around C1-C1´bonds and bonds between radical atoms and rings. The minimum energy geometry found was used as input for subsequent analysis and the corresponding energy values (kJ/mol) for the local minimum geometrical conformers were reported in Table 1. Geometrical rotations around the bonds of the rings and the radicals were categorized as true minima of the potential energy of the surface based on the absence of imaginary vibrational frequencies.

### 3.2. Nicotine Analogs Docking and α7-nAChR Binding Interaction

**Fig. (2).**
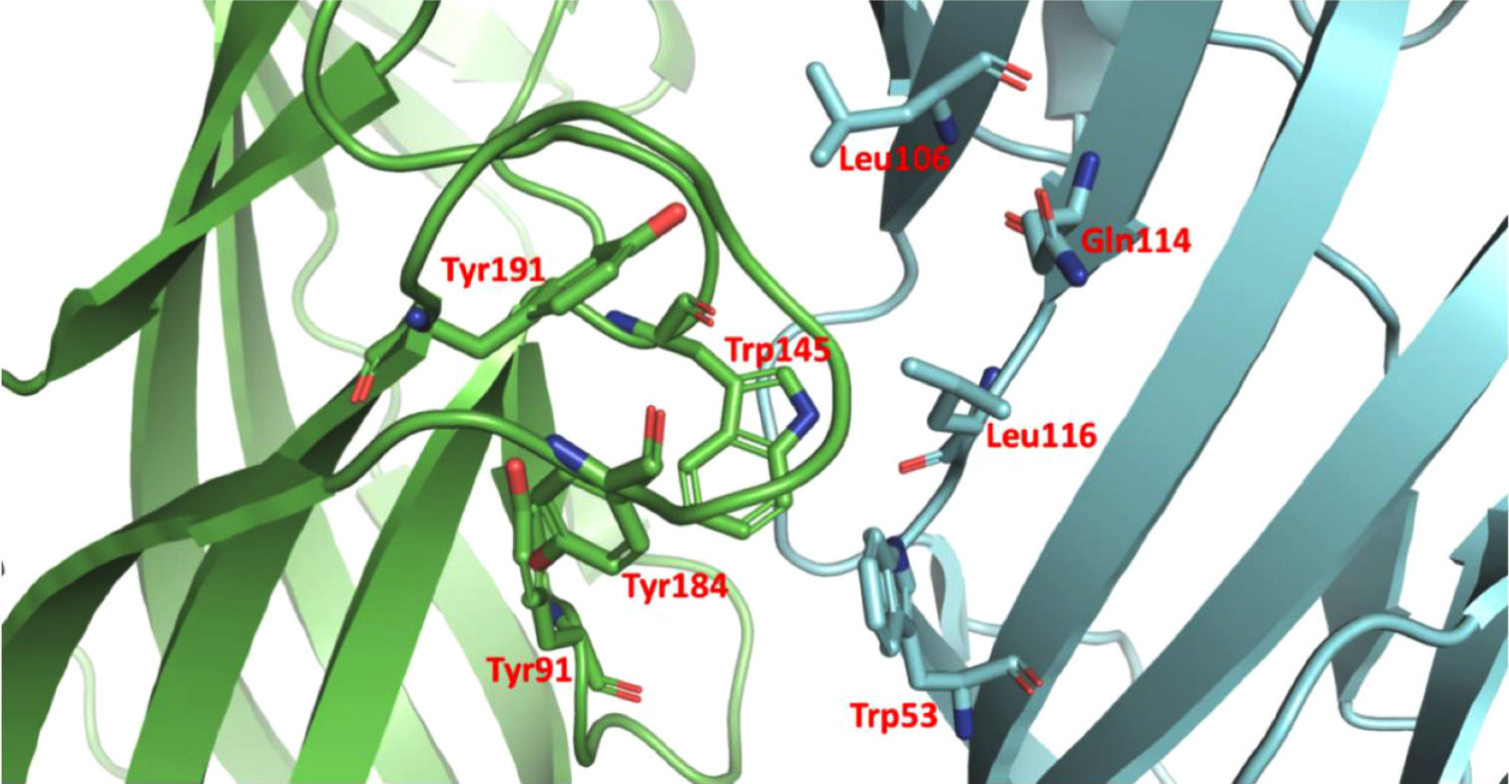
Structural composition of the active site of α7 nAChR. Green and blue represent the two α7 nAChRs subunits composing the active pocket. All α7 nAChRs have 5 identical active sites across the intersections of the α7 homosubunits. Additionally, the localization of the residues composing the active site of the pentameric α7 nAChR is presented highlighting the geometrical orientation.

α7 nAChR has 5 active pockets located at each subunit interactions of the protein complex. Considering that the homopentamer has 5 identical active pockets, we modeled the interaction within one of the pockets considering all the 5 pockets in the homopentamer subunits interaction region are identical. AChBP ligand-binding structure possesses conserved domain residues forming a narrow hydrophobic pocket including A (Tyr91), B (Trp145), C (Tyr184 and Tyr191), D (Trp53), E (Leu106, Gln114 and Leu116). Whereby A, B, and C are present in a different AChBP ligand-binding subunit than D and E (Fig. 2). Following the structural analysis, the nicotine analogs were set to interact with the active pocket within the interaction of the α7 nAChR homo-subunits. Nicotine structure resulted in a geometrical docked conformation in which the pyrrole ring is oriented towards the C loop favoring Van der Waals interactions with amino acids TYR91, TRP145, and TYR184 of the chain A, in addition to LEU106 and TRP53 of the chain B (Table 2).

Docking models were performed with the top ligand conformations and the energy interaction values are presented in Table 3. Evaluated nicotine analogs, except for A1 and A3, have minor binding energy compared with nicotine (Table 3). Energetically minimized protein structures interacting with nicotine and analogs were used to calculate Root-Mean-Square Deviation of atomic positions (RMSD) in a comparative manner against the unbonded receptor and α7 nAChR docked with nicotine (Table 3). Such an unbonded model was set as the minimized structure of α7 nAChR without ligand on the active side.

### 3.3. Clustering of Similarity

Two manually curated groups of compounds with known α7 nAChR agonistic and antagonistic activities were used as input for the cluster. The first group consisted of α7 nAChR agonists SAK3 [43], Nicotine, Acetylcholine, TC-1698 [44], PNU-282987 [45], DMXB [46], and ABT-107 [47]. The second group was composed of receptor α7 antagonists and included methyllycaconitine [48], mecamylamine [49], neostigmine [50], anisodamine [51] and bupropion [52]. To the best of our knowledge, these are all the compounds reported with agonistic neuroprotective activity and agonistic activity over α7 nAChR. In this sense, we set a positive response of PI3K/AKT for the first group and we hypothesized a null activation of PI3K/AKT SP or repression by other molecular mechanisms, and therefore no neuroprotective activity [53]. The same procedure previously mentioned was applied to obtain the optimized conformers for each group and all were categorized considering general descriptors of physicochemical properties [54, 55]. Surprisingly, the dendrogram of similarity was found to be related to the variables and the neuroprotective activity but with inconclusive scores for similarity (Supplementary Fig S1). Nevertheless, cluster values of similarity for PaDEL-Descriptors structural, physical and chemical values were used to enrich the known experimental data of the ligands.

### 3.4. Signaling Pathway Reconstruction and Predictive Model of Interaction-Response

**Fig. (3).**
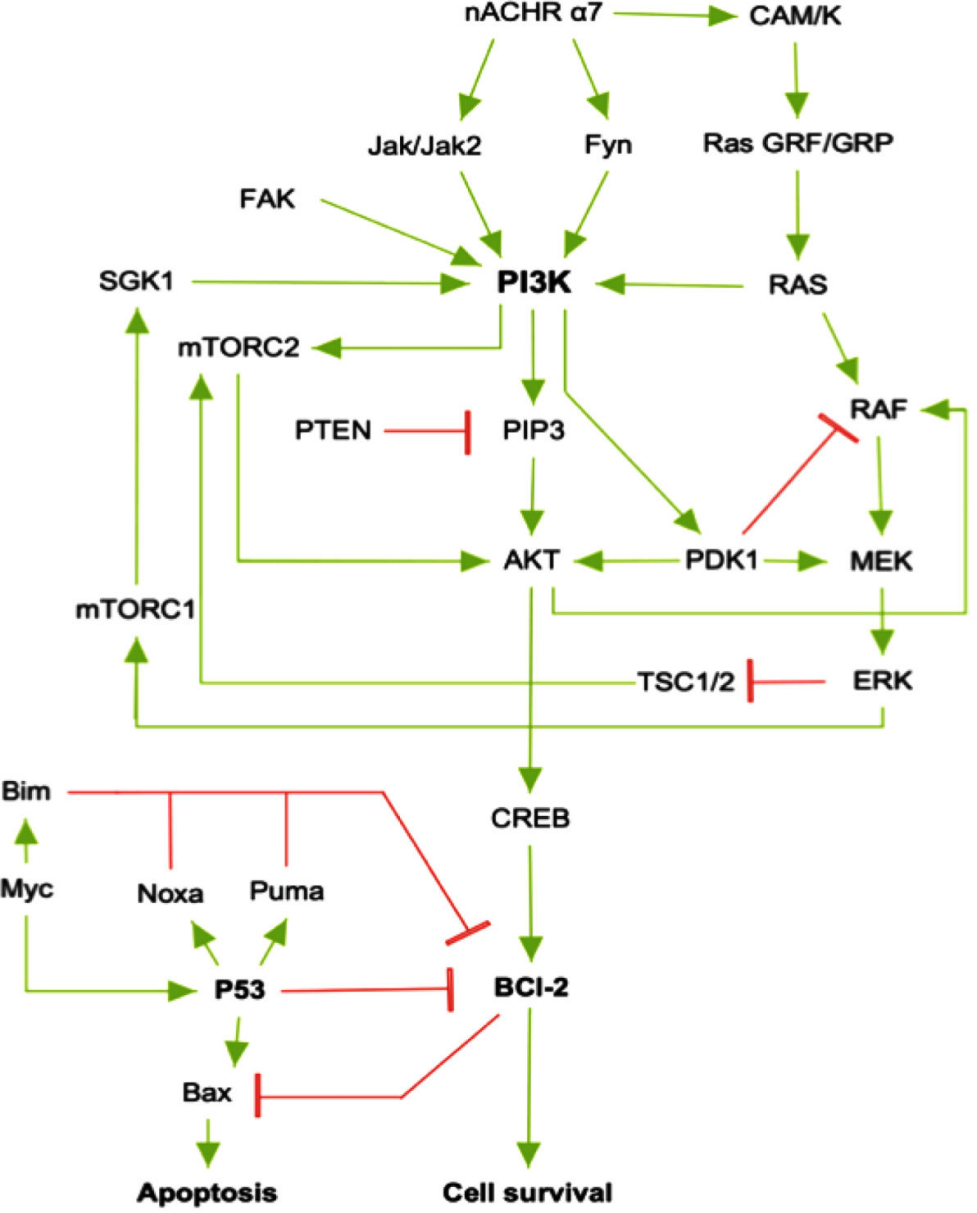
PI3K/AKT Signaling pathway network reconstruction. Each node represents the proteins selected for the SP modulating the activity/expression of Bcl-2. Positive response to proliferation and cell survival was modulated by PI3K/ATK core SP and repression of Bcl-2 was added to the model by including P53. Such a network has a repressive activity over the binomial output. Worth noticing, the network is directional and with feature specific edges, meaning that green edges have a positive modulation of the ending node and red have a negative regulation of the nodes across the network.

The reconstructed model of PI3K/AKT SP presented both activation or inhibition of Bcl-2, as it occurs within the cell [20]. The network is composed of 28 nodes (proteins) that represent relevant components of the pathway (α7 nAChR, PI3K, AKT, CREB, etc), 2 final states (activation/inhibition) and 43 interactions between the nodes (Fig. 3). The network was constructed using nodes with biological relevance associated with PI3K/AKT and P53 proliferative regulatory activity.

With previously mentioned clustering and PaDEL-Descriptors data, 1848 variables were obtained characterizing physico-chemical and pharmacological properties for each one of the molecules, both agonist/antagonist and nicotine analogs. Reducing the number of variables for subsequent analysis, principal component analysis (PCA, Supplementary Fig S2) and K-mean decomposition (Supplementary Fig S3) were used maintaining data variance explanation. First PCA three dimensions explained 98.9% of variance across the original dataset (Supplementary Fig S2) while the K-mean showed a variance explanation of 57.1% for two-dimensions and the corresponding coordinate values (Supplementary Fig S3).

**Fig. (4).**
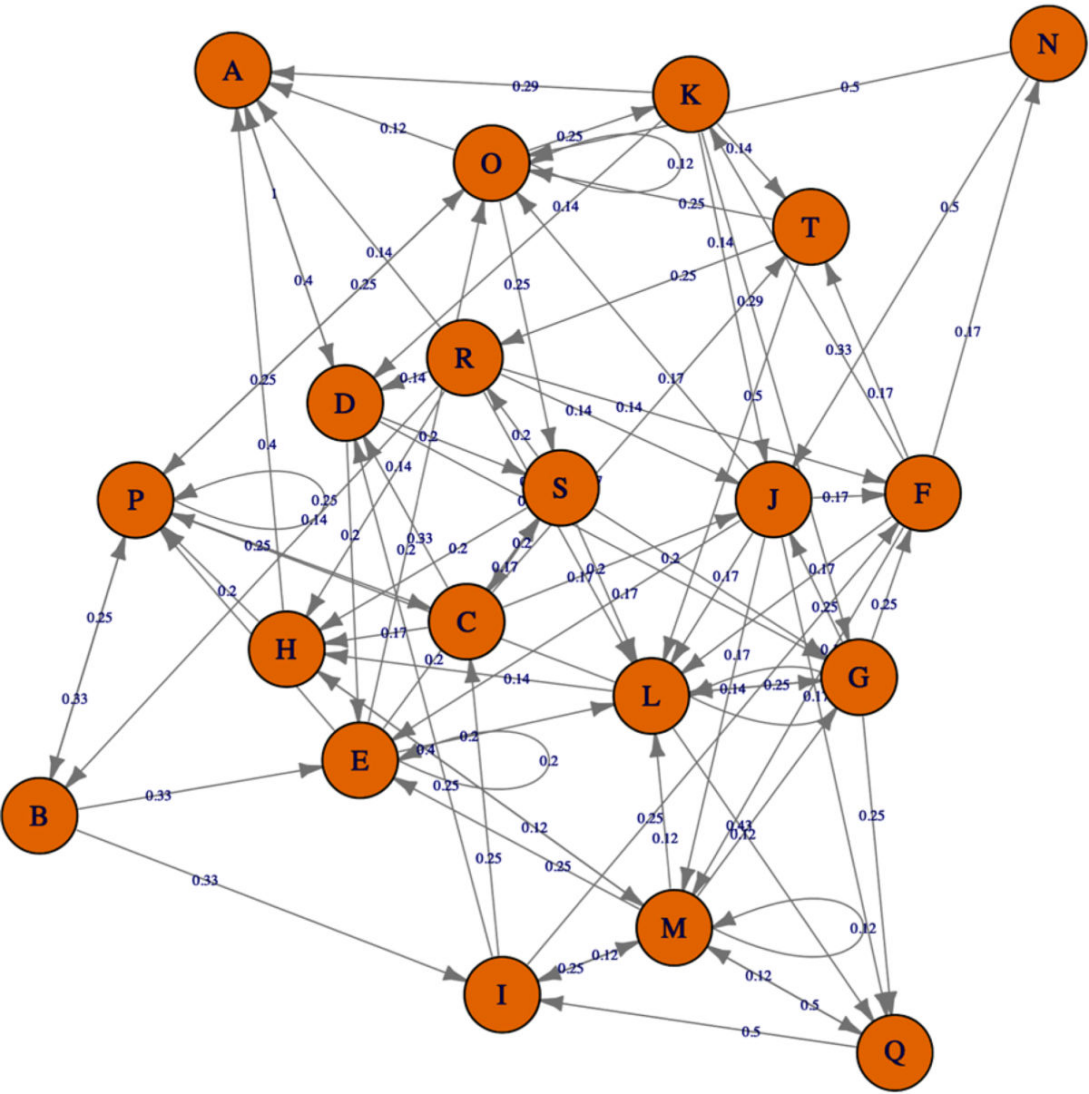
Graphical representation of the probability transitions in the MCMC model of the PI3K/AKT directed network. Optimization was reached when the model stabilizes the probabilities between nodes. In general, each node represents a state involved in the SP and the edges in the graph establish a relationship between the states.

**Fig. (5).**
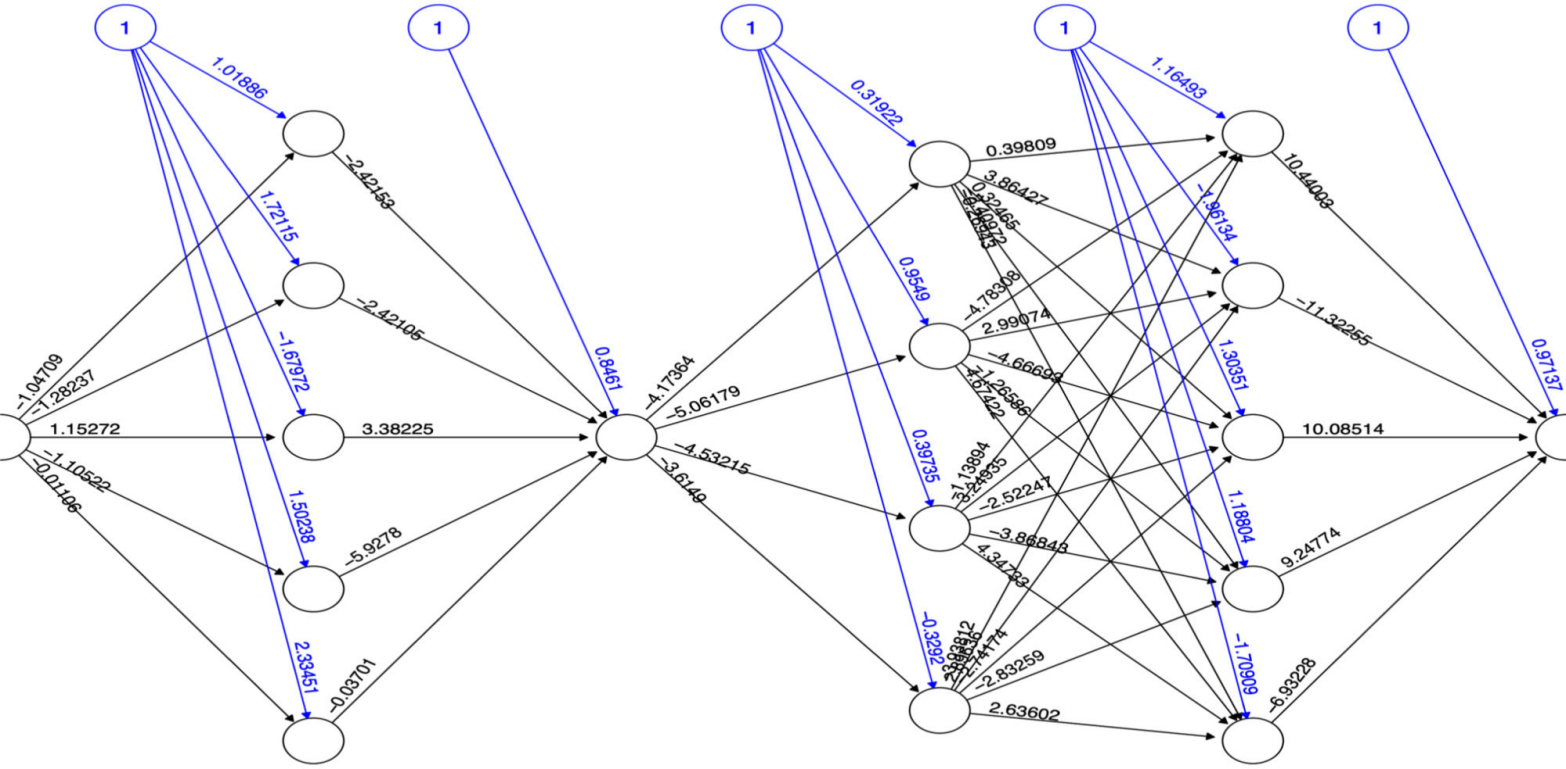
ANN multi-perceptron architecture obtained with the MCMC SP topology. The layers and the number of neurons related to each one of the layers are correlated to the architecture of the MCMC optimal topology. The output of the ANN was set as an activation or repression binomial response.

Based on the PI3K/AKT architecture the resulting MCMC topology representing probabilities of transition within the network (Fig. 4). In this case, the resulting Markov Chain Monte Carlo (MCMC) model was able to quantify the transitions between the directed network resulting in a model capable to determine the ANN architecture. The most effective number of hidden layers of the ANN was determined by iterative topology generation and convergence was reached at 100.000 iterations. Such an approach resulted in a multi-perceptron ANN with 4 hidden layers with 5, 1, 4 and 5 nodes respectively (Fig. 5). In general, the resulting topology lack a canonical hidden layer architectures considering these models tend to reduce the number of nodes per layer across the model [56].

Training datasets from the PCA and K-mean were evaluated for each of the ANN learning with 1000 iterations with randomized series and reported values of misclassification (Table 4). Backpropagation with weight tracking coupled with PCA model was capable of generating consistent results. In this sense, the consistency of resilient backpropagation with weight backtracking and PCA was 100% but resilient backpropagation without weight backtracking and K-mean showed 98%. These results suggest that both methods were able to predict the binomial output for the training and testing datasets. Nevertheless, throughout the iterations not all the predictions were identical in resilient backpropagation without weight backtracking and K-mean.

The resulting best ANN model implementing resilient backpropagation with weight backtracking is based on the evolving adaptation rule with a learning process based on error function [57].

(A) $$\Delta_{ij}^{\left(t\right)}=\{\begin{array}{c} \eta^{+}*\Delta_{ij}^{\left(t-1\right)},\;\mathrm{if}{\frac{\partial E}{\partial \omega_{ij}}}^{\left(t-1\right)}*{\frac{\partial E}{\partial \omega_{ij}}}^{\left(t\right)}>0\\ \eta^{-}*\Delta_{if}^{\left(t-1\right)},\;\mathrm{if}\;{\frac{\partial E}{\partial \omega_{ij}}}^{\left(t-1\right)}*{\frac{\partial E}{\partial \omega_{ij}}}^{\left(t\right)}<0\\ \Delta_{ij}^{\left(t-1\right)},\;\mathrm{else} \end{array}\;\;\text{where}\;0<\eta^{-}<1<\eta^{+}$$

(B) $$\Delta \omega_{ij}^{\left(t\right)}=\{\begin{array}{c} -\Delta_{ij}^{\left(t\right)},\;\mathrm{if}\;{\frac{\partial E}{\partial \omega_{ij}}}^{\left(t\right)}>0\\ +\Delta_{ij}^{\left(t\right)},\;\mathrm{if}\;{\frac{\partial E}{\partial \omega_{ij}}}^{\left(t\right)}<0\\ 0,\;\mathrm{else} \end{array}\;\;\omega_{ij}^{\left(t+1\right)}=\omega_{ij}^{\left(t\right)}+\Delta \omega_{ij}^{\left(t\right)}$$

and

(C) $$\Delta \omega_{ij}^{\left(t\right)}=-\Delta \omega_{ij}^{\left(t-1\right)},\;\mathrm{if}\;{\frac{\partial E}{\partial \omega_{ij}}}^{\left(t-1\right)}*{\frac{\partial E}{\partial \omega_{ij}}}^{\left(t\right)}<0$$

Briefly, for the adaptation rule each update-value $\Delta_{ij}^{\left(t\right)}$ changes depending on the sign for each partial derivative of *ù*_*ij*_ at point *t* (A). If the values are too big then the algorithm skips a local minimum and the function decreases by $\bar{\eta }$. Once $\Delta_{ij}^{\left(t\right)}$ adapts, $\Delta \omega_{ij}^{\left(t\right)}$ changes according to $\frac{\partial E}{\partial \omega_{ij}}$ results (B). Positive values of the derivative mean an error increase; therefore, the function is decreased by its $\Delta_{ij}^{\left(t\right)}$. Finally, the exception presented deals with changes in partial derivative sign (C) [57]. In that case, the step was too large and missing the minimum, so the previous $\Delta \omega_{ij}^{\left(t\right)}$ is reverted. Finally, with the best trained structure method with PCA and Resilient backpropagation with weight backtracking and the MCMC derived ANN multi-perceptron architecture it was possible to predict the neuroprotective potential of the nicotine analogs. This model predicted A1, A2, A3, A4, A6, A7 and A8 as potential neuroprotective agents. In this aspect, our model predicted that 7 out of 8 analogs had putative neuroprotective activity due to the association of the binomial output of the ANN with the activation of Bcl-2.

## 4. DISCUSSION

### 4.1. Conformer Geometry and Ligand-Receptor Interaction

Even though research efforts have been done regarding the pharmaceutical potential of nicotine analogs, little assessment of some critical aspects of their conformational variability has been studied [15, 58]. Recently, the crystallographic structure of the extracellular domain of the nicotinic receptor α7 in complex with epibatidine was reported [59]. Although interactions described for the complex agree with our results, it was found that THR146, CYS187, and CYS186 could also be involved in the protein complex due to molecular proximity. Docking with analog A5 had a high-affinity score (−4.7) with respect to nicotine (−5.5) (Table 3). A5 affinity can be attributed to the geometrical conformation establishing hydrogen bonds and radicals orientation towards TRP145, similar to epibatidine. The high affinity of epibatidine has been related to large inter-nitrogen distance previously reported as 4.6 Ǻ [34].

Considering H-bond acceptors and donors number, analogs and nicotine had similar values. Specifically, A2, A3, A4 and A7 have two H-bond donors in comparison with three for A1, A5, A6 and A8. In terms of routable bonds, A5 and A6 differ from the single bond in nicotine considering the presence of radicals across the pyrrole ring (Table 1). Fluorine radical of A5 and the methyl group attached to the oxygen radical of A6 are rotation-capable bonds relative to the ring plane. Distinct similarity values in the clustering analysis for analogs A3 and A8 (Supplementary Fig S1) are not reflected in the values of energy affinity or interacting residues. Interestingly, A3 and A8 are the only analogs with a single methyl group addition to the pyrrolidine ring. In this sense, the group generated between A3 and A8 can be associated with the presence of the methyl group and the double bond with nitrogen in the pyrrolidine ring. Nevertheless, clustering was capable of generating patterns based on atom pair descriptors and molecular fingerprints, the model is inefficient to replicate the docking data. All analogs clustered with nicotine and TC-1698 with a node value of 0.4 in the dendrogram, but A3 and A8 showed significant similarity with TC-1698, suggesting agonistic and neuroprotective activity. Interestingly, A3 and A8 also have a null net charge of interaction with the protein (Table 3). Nevertheless, further *in vitro* and *in vivo* experiments are necessary to assess the neuroprotective capacity of the tested molecules and *in silico* modeling must be performed to establish a correlation between structural signatures and biological activity.

Besides affinity energy, the type and amount of interactions are crucial to determine the role of the docking in the protein function [13]. In this case, nicotine interacts with TRP53, Tyr91, Leu116, Trp145, Tyr184, Cys186, Cys187 and Tyr191 through hydrophobic interactions and without hydrogen bonds (Table 2). Compared with nicotinic receptor α7 in complex with epibatidine interaction, only residues Leu106 and Gln114 were absent in the interaction [60]. Compared with the analogs, hydrophobic interactions with Leu106 were present in the analogs A1, A5, A6, and A8, but no hydrophobic interactions or hydrogen bonds were found with Gln114 (Table 2). The rest of the residues interacting with nicotine were found interacting at least once with any analog in similar geometrical orientations. Interestingly, none of the analogs presented the same interactions as nicotine, but the energy changes were not higher than 0.4 kJ/mol, except for A5 with 0.8 kJ/mol. Such values of A5 could be attributed to the highly electronegative fluorine radical interacting with Thr146. An increase in Affinity energy could be associated with the presence of hydroxyl radical in the polar sidechain. Moreover, fluorine has been associated with secondary effects in humans and, if consumed in higher doses, has been reported to be toxic, thus suggesting that A5 is not a suitable neuroprotective agent [61, 62]. However, further studies must be performed in order to identify the role of specific residues in the activity of the complex and specific analog modifications effect over protein interactions.

Additionally, taking into account the comparison of RMSD values, after depuration of 25% of the misplaced atoms, unbonded protein and interacting complex with nicotine were lower than 0,1. Nicotine and analogs with the unbonded protein, RMSD validated our docking result (Table 3). RMSD for A2, A3 and A8 are higher than the rest of analogs when compared with the bounded complex with nicotine meaning that the geometrical modifications of the protein are higher. In this sense, A3 and A8 have the potential of interacting with the receptor similar to other neuroprotective compounds but changing the receptor response when compared with nicotine. The similar biological activity could be deduced also by considering the residues interacting, A3 and A8 as well as A6 and A7 present hydrogen bond interactions similar to nicotine (Table 2). With the structural and geometrical analysis is possible to conclude that all analogs except for A5 have neuroprotective potential and that A3 and A8 have an interesting behavior that must be addressed with *in vitro* and *in vivo* experiments. For A5, we present evidence of difference in energy affinity, interacting residues and fluoride radicals lead to the potential toxicity of A5.

Nevertheless, inconclusive relations between ligand structure, complex bonding and neuroprotective response occurred within the clustering. Even though consistent aggrupation occurred for all the neuroprotective compounds, antagonists generate a disperse distribution in the graph. The clustering FOF method was able to discriminate between biological effects but only strong aggrupation occurred only for neuroprotection positive molecules (Supplementary Figure S1). Acetylcholine was discarded considering divergence and ubiquitous neurotransmitter distribution coupled with no associated neuroprotective function [63]. Worth noticing, the potential toxicity of the other analogs should be addressed by experimental methods considering no information is available in cell culture or animal models neither *in silico* testing

### 4.2. Neuroprotective Prediction Modeling

Only a few studies have employed ANN in the study of signaling pathways [42, 64]. For instance, a 3-layered multi-perceptron with backpropagation learning and sigmoid activation function was implemented across 96 genes to identify biomarkers in children sarcomas [64]. Moreover, a simplified neural network comprising two microenvironmental input nodes (growth factor and death signal), and two phenotype output nodes (pro-growth and pro-death) was used to integrate environmental and molecular characteristics of cancer progression [42]. However, to the best of our knowledge, this is the first study that combines structural data, docking simulations, MCMC and ANN evaluating the modulation of PI3K/AKT allowing the prediction of neuroprotective new compounds. By including a higher set of variables, analogs cluster near to nicotine for both PCA and K-mean decomposition analysis (Supplementary Figs 2 & 3). Nevertheless, the absence of strong groups in the PCA and K-mean analysis showed that synthetic data was not conclusive to predict the biological activity by themselves. With this in mind, the ANN model was essential to discriminate between the groups and underline the fundamental properties of the analogs based on the SP structure, using both PCA and K-mean synthetic values

SP are dynamic systems in which crosstalk with neighbor paths is essential to modulate signal intensity [65]. P53 negative feedback increased the robustness of the model by modifying Bcl-2 binomial output and information flow across the model supporting network regulation (Fig. 3). Additionally, ionotropic channels, such as α7-nAChR acting over PI3K/AKT lead to an activation without explicitly acknowledging the presence of JAK [14]. MCMC PI3K/AKT-derived model considered a matrix of transition representing network structure and probabilities of transition across PI3K/AKT leading to robust computational inferences [66]. By applying MCMC to the network topology it was possible to transfer the biological structure of the network to a quantitative model capable to modulate ANN architecture. The resulting non-canonical multi perceptron structure resulted as biological-based discrimination of ANN architectures through iterations using the MCMC matrix. Similar approaches such as the *drop-out* technique allow to drop units from the neural network during training hence preventing an excessive coadaptation and overfitting of the network [12]. Iterative processes converged at the minimal topology with a significant predictive capacity of the ANN model reducing layer and nodes number [67]. The hidden layers were locked according to the MCMC product (Fig. 4), ensuring that the subsequent topology represented the biological information contained in the SP. In this aspect, the synthetic iterative randomized data sets integrated with dimensional minimization and ANN, allowed the determination of the best predictive method reducing error and ensuring consistent predictions during training.

PCA and K-mean dimensional reduction showed to be an effective way to effectively decrease sample variability to a low number of synthetic values [68]. By reducing the number of variables from 1848 variables to a small non-autocorrelated dataset, PCA and K-mean reproduced clustering results. This approach was implemented in order to optimize the performance of the strategy and reduce overfitting of the trained ANN model. Through variable reduction, we increased the sensibility and reliability of the model to predict the binomial neuroprotective signal output. This data was used as training/testing for the ANN model evaluating some of the developed learning algorithms [69]. Identical split differences in misclassification for smallest absolute derivative, backpropagation and smallest learning rate with PCA and K-mean, and resilient backpropagation with weight backtracking with K-mean were identified (Table 4). Improvement was found for 25% misclassification for both resilient backpropagation with weight backtracking with PCA and resilient backpropagation without weight backtracking with K-mean. With this last method inconsistency in the predicting output was found meaning the output change through 2% of the iterations. Resilient backpropagation with weight backtracking with PCA resulted as the best combination for neuroprotection prediction, considering 100% of the predictions were consistent along with the iterations. The resulting model was based on the implementation of an adaptation rule with a learning based on error values [57]. By predicting the binomial neuroprotective output of the ANN with synthetic PCA data from structural conformational, docking analysis and physicochemical featuring, made possible to determine that A1, A2, A3, A4, A6, A7 and A8 have potential neuroprotective agents. In general, the results are consistent with the experimental data available for some of the compounds [10, 47 – 52].

Even though this model considers the main aspects of SP response to external stimuli, some key considerations are necessary. Structurally, the number of reference compounds used was low, making it necessary to improve α7-nAChR agonist/antagonist data through identification and screening. Data restriction can lead to predicting bias of the model, overfitting or fixed prediction. In this matter, we recommend implementing both agonist and antagonist *in silico* and experimental screening to increase the training dataset. Although, the inclusion of synthetic molecules, such as SAK3, PNU-282987 and allosteric modulators allowed to improve predictions considering previously reported induction of neuroprotection through the modulation of PI3K/AKT/Bcl-2 [14]. Furthermore, the model can be used to address the secondary effects of nicotine analogs by increasing the dataset with addiction/reward molecules like nicotine and methyllycaconitine [48].

Correcting inner functions for noisy data needs to be coupled with further testing, essential to determine the true potential of nicotine analogs and antagonists for PD therapy. In this regard, we propose a mathematical *in silico* model that has to be used integrated with *in vitro* and *in vivo* data, either on dopaminergic neuronal cell lines, cerebral organoids or animal models. Database cluster enrichment with experimental data allows to increase the reference compounds and therefore reducing possible prediction bias and overfitting. Even so, the model strategy was in accordance with previously published experimental data [10, 47 – 52] proving to be robust enough as a testing method for further research. Worth noticing, SP are dynamic systems, therefore we suggest that continuous parameters need to be addressed to consider essential intermediate states [65]. Finally, this strategy opens the door to improve neuroprotection prediction in the future by integrating protein dynamics, partial differential equations and other machine learning approaches. These type of approaches are necessary to improve drug scanning efficiency in PD and any other disease founded upon SP.

### 4.3. Conformational Analysis and Protein Structure Preparation

8 novel nicotine analogs (Table 1) were used in the present study: (3R,5S)-1, methyl-5-(piridine-3-yl) pirrolidine-3-ol (A1), 3-(1,3-dimethyl-4,5-dihidro-1h-pirazole-5-yl) piridine (A2), 3-(3-methyl-4,5-dihidro-1h-pirazole-5-yl) piridine (A3), 3(((2S-4R)-1,4-dimethylpirrolidine-2-yl)) (A4), 3-((2S,4R)-4-(fluoromethyl)-1-methylpirrolidine-2-il)piridine (A5), 3-((2S,4R)-4-methoxi-1-methylpirrolidine-2-yl) piridine (A6), 3-((2S,3S)-1,3-dimethylpirrolidine-2-yl) piridine (A7) and 5-methyl-3-(piridine-3-yl)-4,5-dihidroisoxazole (A8). Molecular structures were sketched on Avogadro using MMFF94s forcefield, correcting atom type and chirality [70]. Calculations were carried out with Gaussian 16 [71], using B3LYP level of theory and cc-PVDZ basis set. In order to find the minimum energetic conformation, the structures were optimized at the DFT B3LYP/6–31G level. The conformational analysis was carried out on minimized rotating bonds between pyrrole, derivate rings, and pyridine rings, including the bonds of all the radicals of the pyrrole ring for each ligand. The maximum number of conformers for each molecule was set to 30, with the 10 lowest energy conformations used for the docking simulations.

The crystal structure of α7-nAChR in complex with lobeline was obtained from the RCSB Protein Data Bank (PDB ID: 5AFJ) [60]. AutoDockTools [72] was used to assign polar hydrogens and add Gasteiger charges. The geometry of the receptor was optimized using the MM2 molecular mechanics force field. The neutral and ionized states of aliphatic amine and carboxylic acid groups of compounds to be docked were protonated and deprotonated separately.

### 4.4. Molecular Docking

To determine the interaction between the studied molecules and the receptor, docking simulations were performed with AutoDock4 (version 4.2) [72]. The active site of the pentameric structure of α7-nAChR was defined as the interfaces between subunits that were within 12 Å from the geometric centroid of the ligand [60]. Within the 3 domains of the protein (extracellular, intracellular and transmembrane), the pocket is located in the extracellular side of the receptor with residues from loops A-C of the principal subunit and loops D-E of the complementary subunit. Default settings for small molecule-protein docking were used throughout the simulations.

Docked conformations were clustered according to the interacting energy combined with geometrical matching quality. The complexes with the best score were taken as the lead conformation for each compound. The correlation between key interactions obtained from the computational simulations and the interactions reported by the crystallographic structure of α7-nAChR in complex with lobeline was further explored. To further analyze the differences between the interaction of the analogs and the receptor, we calculated the RMSD values between analogs, nicotine and the receptor. RMSD was calculated using 85% of the atoms resulted after 3 outlier rejection cycles. The energetically minimized docked structures of the α7-nAChR receptor were made with Chimera [73], using 10000 steepest descent steps, 0.001 Ǻ steepest descent steps size, 10 conjugate gradient steps, 0.001 Ǻ conjugate gradient steps size, update per 10 intervals, gasteiger method for charge and Amber ff14sb algorithms for residues.

### 4.5. Physicochemical Clustering and Dimensional Decomposition

To determine the features for each molecule and their structural similarity, physicochemical property predictions, classification and clustering of structures were performed on ChemmineR, R package [54]. Structural similarities between analogs were compared to a manually curated dataset of 7 agonists and 5 antagonists of α7-nAChR with reported neuroprotective activity [43 – 52]. This method allowed us to increase the size of the available data, robustness and reproducibility. In the dataset, agonists of α7-nAChR were associated with the induction of a neuroprotective pathway, either expressed through the induction of cell proliferation or apoptotic prevention. Antagonists were set to block either the proliferative activity by PI3K/AKT of the signaling pathway or the associated Ca^2+^ mobilization of the receptor [51, 52, 74, 75].

Clustering analysis was based on molecular descriptors, such as molecular formula, molecular weight, atom frequency and functional groups. To determine the optimal cluster, a matrix of molecular descriptors for all molecules was used, categorizing the ligands according to the structural, physical and chemical variables. To increase the robustness of the clustering model, additional physicochemical data from *de-novo* featurization was included [55]. To ensure a proper physicochemical featurization and increase the number of variables available for further analysis, data from *de-novo* characterization using PaDEL-Descriptors [55] was also included. Dimensionality reduction of the dataset was done by principal component analysis (PCA) and k-mean decomposition. The mentioned approaches were used to identify the multiparametric data capable to describe the agonistic and antagonistic function and the relation between the activity and structure of the ligands in the dataset.

### 4.6. Signal Reconstruction

To establish a reliable topology of the SP related with the neuroprotective capacity of nicotine, a manually reconstructed network for the PI3K/AKT SP was developed using information from KEGG [76 – 78] and PantherDB [79]. To ensure the biological coherence of the network, our model was enriched with additional protein-protein interactions that are present in the PI3K/AKT/mTOR interactive pathway [80]. Finally, this PI3K/AKT SP was integrated with a manually curated model of p53 to generate a negative feedback over Bcl-2. In this aspect, the model included 6 specific proteins (Myc, Bim, P53, Bax, Noxa, Puma) associated with the partial inhibition of the Bcl-2, which emulates the SP biological behavior [66].

### 4.7. Activity Prediction and Interaction-Response Model

In order to elucidate the quantitative relationship between α7-nAChR receptor and the activation of Bcl-2, a Markov Chain Monte Carlo (MCMC) model for the whole network was implemented. This model was based on the reconstructed PI3K/AKT SP network and was generated using the R package *MCMCpack* [81]. A matrix of transitional stages predicting the most suitable path across the nodes was associated with the end stages of the MCMC that represented Bcl-2 expression as a binomial logical argument. The construction of the Artificial Neural Network model (ANN) was performed with the *NeuralNet* package in R [82]. To determine the canonical architecture for the ANN we used an optimization algorithm using 100.000 iterations using the MCMC transition matrix as an input. A stable multi-perceptron structure was set to have a minimal number of hidden layers with the capacity of representing the MCMC transition matrix, as shown below:

Briefly, a matrix *x* is used as input in the first layer of the multiperceptron. Each node of the hidden layer computes *f*_*(x)*_ to finally generate a binomial *y* output.

The dimensional reduction methods were used to train the ANN using the coordinates in the PCA and K-mean. One thousand randomized training datasets were selected and associated with the binomial activity of the ligand dataset. To optimize the predictive capability of the model using the training datasets, four ANN algorithms were compared: Backpropagation with a learning rate of 0.001, Resilient backpropagation with weight backtracking, Resilient backpropagation without weight backtracking, smallest absolute derivative model and smallest learning rate. Additionally, for each possible combination of dimensional minimizations and ANN methods, the random data subsets used as training were iteratively generated, after excluding the testing values. To ensure the robustness of the prediction, values for misclassification error were obtained for each training and testing combination. In this aspect, the best model acquired was set to minimize the error to reduce the amount of false-positive predictions.

## CONCLUSION

We present a novel computational strategy as a novel quantitative approach used to predict the potential neuroprotective activity of 7 nicotine analogs in the context of PD therapeutics (A1, A2, A3, A4, A6, A7 and A8). To the best of our knowledge, this is the first machine learning neuroprotective computational strategy for ligand activity prediction over α7-nAChR activating PI3K/AKT/Bcl-2. By conformational analysis, molecular docking, the use of PaDEL variables and geometrical optimization, we obtained robust evidence suggesting nicotine analogs activity compared with nicotine.

The model also showed the potential of ANN integrations with a dimensional reduction to avoid overfitting by autocorrelation. Resilient backpropagation with weight backtracking with PCA showed to be the best combination for ANN training to validate the neuroprotective activity. Nevertheless, additional data is needed to ensure model robustness, decrease the error of misclassification and avoid fixing predictions. Nicotine analogs have promissory future as candidates for PD treatment if future improvements to these strategies are performed. Nevertheless, further studies must be performed to identify the potential of nicotine analogs as neuroprotective compounds and the application of these methods in neurodegenerative research.

## Supplementary Material

Supplemental material

## Figures and Tables

**Table 1.** Nicotine and nicotine analogs studied. 2D structure of each one of the ligands and energy values of each compound (kJ/mol) are presented. Structural similarity with nicotine was based upon energy values and the pyridine and pyrrolidine rings present in their structures. Nicotine analogs have modifications across the pyrrolidine ring of the nicotine backbone focusing in the addition of methylations, hydroxyl groups or highly electronegative elements. All the corresponding structures were provided by the laboratory of chemistry at the Pontificia Universidad Javeriana. Further analysis of all nicotine analogs will reference the ID provided ranging from A1 to A8.

Molecule	IUPAC Name	Structure	Minimum Energy Value (kJ/mol)
Nicotine	Nicotine	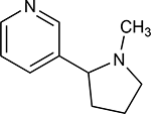	105.636
A1	(3R,5S)-1, methyl-5-(piridine-3-yl) pirrolidine-3-ol	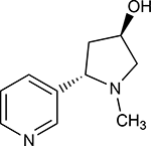	199.330
A2	3-(1,3-dimethyl-4,5-dihidro-1h-pirazole-5-yl) piridine	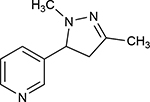	120.593
A3	3-(3-methyl-4,5-dihidro-1h-pirazole-5-yl) piridine	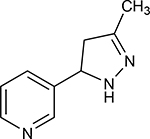	82.944
A4	3(((2S-4R)-1,4-dimethylpirrolidine-2-yl))	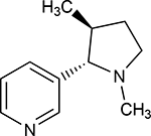	130.095
A5	3-((2S,4R)-4-(fluoromethyl)-1-methylpirrolidine-2-il)piridine	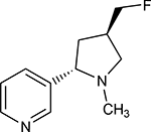	111.131
A6	3-((2S,4R)-4-methoxi-1-methylpirrolidine-2-yl) piridine	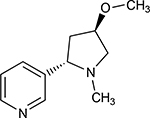	216.058
A7	3-((2S,3S)-1,3-dimethylpirrolidine-2-yl) piridine	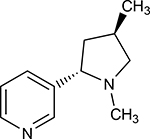	127.645
A8	5-methyl-3-(piridine-3-yl)-4,5-dihidroisoxazole	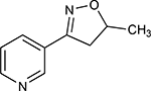	174.670

**Table 2.** Diagrammatic representation of receptor interaction, residues involved in the receptor interaction with analogs and nicotine. Dark shaded cells mean the presence of the interaction between the ligand and the receptor. Discrimination between hydrogen bonds and hydrophobic interactions are shown.

Molecule	Hydrogen Bonds				Hydrophobic Interactions									
	Tyr91	Thr145	Tyr184	Tyr191	Trp53	Tyr91	Leu106	Leu116	Trp145	Thr146	Tyr184	Cys186	Cys187	Tyr191
Nicotine														
A1														
A2														
A3														
A4														
A5														
A6														
A7														
A8														

**Table 3.** Energy and RMSD values for the molecular docking between analogs and nicotine. For RMSD there is a differentiation between α7 nAChRs interacting with nicotine and α7 nAChRs (unbonded) receptor without ligand. For the chimera optimization, the charges for the steepest descent minimization of the ligand were manually added. RMSD values represent the average distance between atoms, in this case, backbone atoms, between protein structures. The lower the RMSD value, the more geometrical similarity between the proteins.

Molecule	Charge of Interaction	Energy of Interaction (kJ/mol)	RMSD	
			Unbonded	Nicotine
Nicotine	+1	−5.5	0,049	-
A1	+1	−5.6	0,069	0,033
A2	0	−5.5	0,013	0,054
A3	0	−5.6	0,039	0,075
A4	+1	−5.1	0,053	0,014
A5	+1	−4.7	0,052	0,030
A6	+1	−5.5	0,047	0,016
A7	+1	−5.5	0,051	0,020
A8	0	−5.5	0,020	0,052

**Table 4.** Values of misclassification error for all different ANN methods and dimensional minimization approaches. Normalized data is shown (0–1).

Learning algorithm	Dimensional reduction method	
	Principal Component Analysis	K-mean
Backpropagation	0.5	0.5
Resilient backpropagation with weight backtracking	0.25	0.5
Resilient backpropagation without weight backtracking	0.375	0.25
Smallest absolute derivative	0.5	0.5
Smallest learning rate	0.5	0.5

## LIST OF ABBREVIATIONS

PD: Parkinson’s Disease
DN: Dopaminergic Neurons
PI3K/AKT: Phosphatidylinositol-4,5-bisphosphate 3- kinase/Protein kinase B
α7-nAChRs: alpha7 nicotinic Acetylcholine Receptor
MCMC: Markov Chain Monte Carlo
PCA: Principal Component Analysis
ANN: Artificial Neural Networks
SP: Signaling Pathway
