# Supplementary material for: Machine Learning Neuroprotective Strategy Reveals a Unique Set of Parkinson Therapeutic Nicotine Analogs
Source: Open Bioinforma J. Author manuscript; Available in PMC 2021 Apr 28. (PMC8081347)
Supplement: Supplemental material [file NIHMS1692257-supplement-Supplemental_material.pdf]

1875-0362/20 *Send Orders for Reprints to* reprints@benthamscience.net

The Open Bioinformatics Journal
Supplementary Material
Content list available at: https://openbioinformaticsjournal.com

Machine Learning Neuroprotective Strategy Reveals a Unique Set of Parkinson Therapeutic Nicotine Analogs

Felipe Rojas-Rodríguez[1,*], Carlos Morantes[2], Andrés Pinzón[3], George E. Barreto[4], Ricardo Cabezas[1], Leonardo Mariño[5] and Janneth González[1]

[1]*Departamento de Nutrición y Bioquímica, Pontificia Universidad Javeriana. Bogotá D.C, Colombia*
[2]*Departamento de Biología, Universidad Nacional de Colombia. Bogotá, Colombia*
[3]*Instituto de Genética, Universidad Nacional de Colombia, Bogotá, Colombia*
[4]*Department of Biological Sciences, University of Limerick, Limerick, Ireland*
[5]*National Center for Biotechnology Information, National Library of Medicine, National Institutes of Health, 8600 Rockville Pike, Bethesda, MD20894, USA*

Article History	Received: September 27, 2019	Revised: December 02, 2019	Accepted: December 04, 2019

SUPPLEMENTARY FIGURES

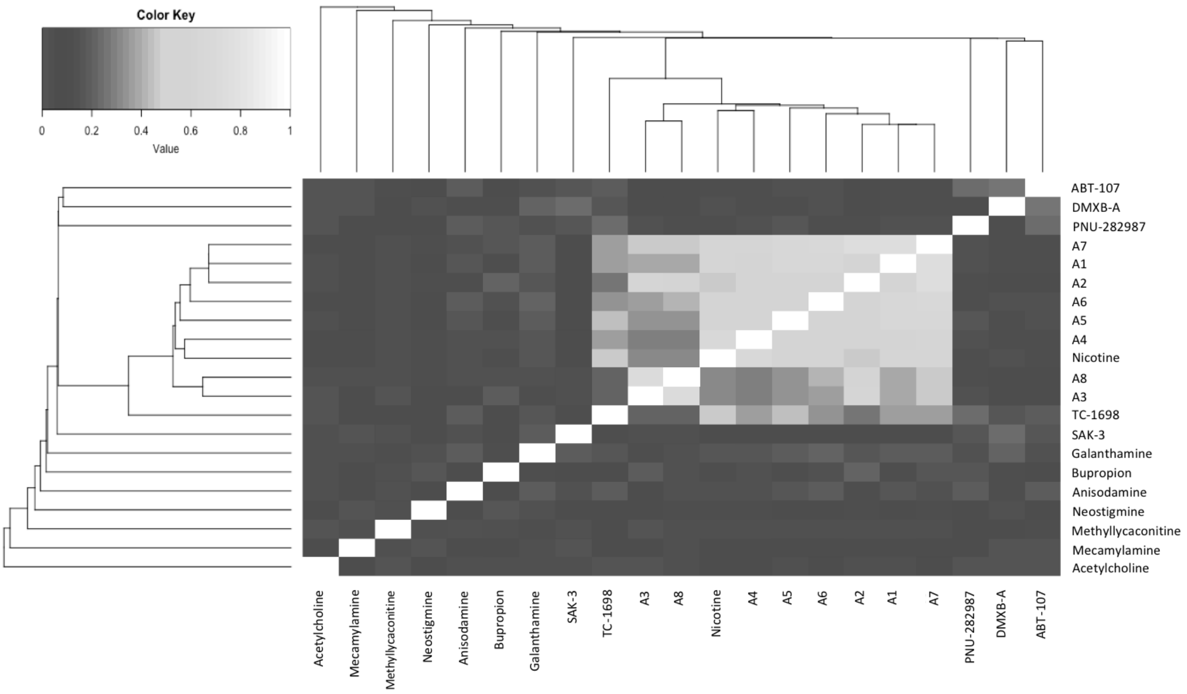

Supplementary Fig. (1). Similarity of molecules related to their potential neuroprotective response. The central cluster of the heatmap contains the analogs of nicotine, nicotine and TC-1698, both related to a neuroprotective *in vitro* positive activity over α7 nAChR.

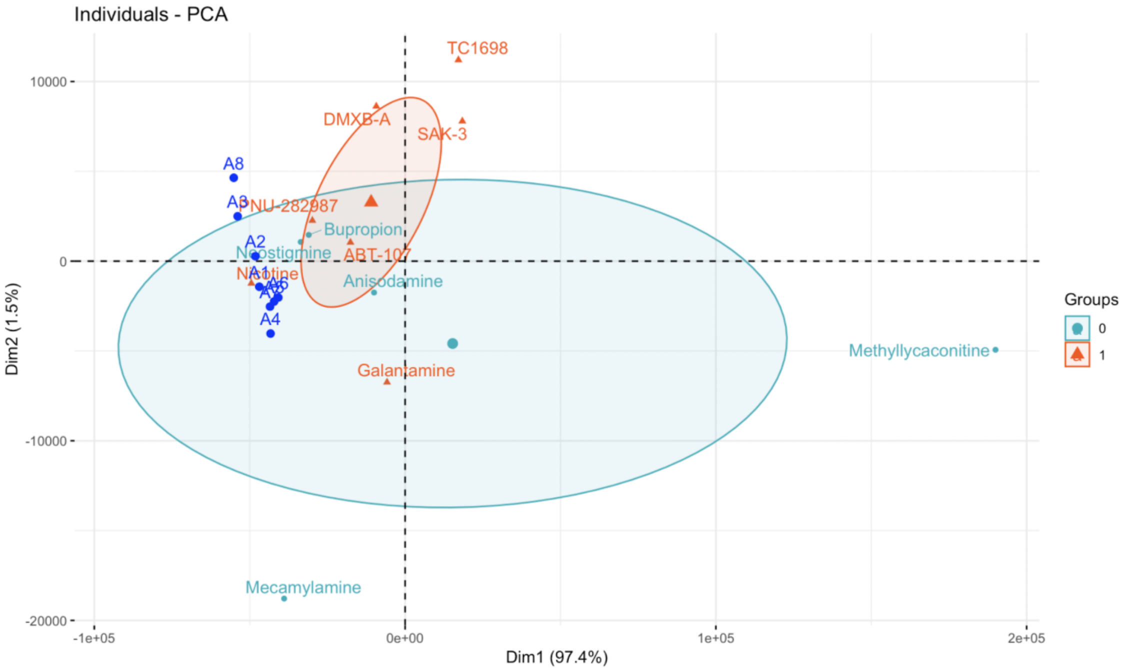

Supplementary Fig. (2). Principal component analysis of the manually curated dataset. 0 represents the antagonist function over the receptor (no neuroprotection) and 1 indicates the molecules related with a positive response of α7 nAChR and putative induction of PI3K/AKT Bcl-2.

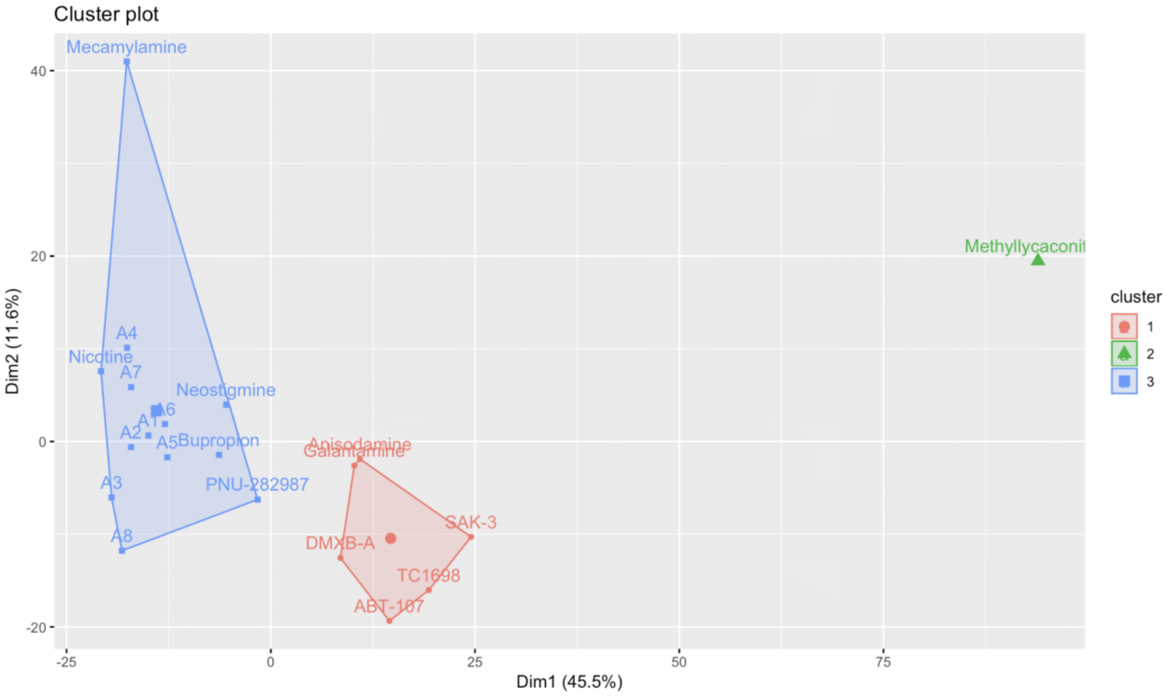

Supplementary Fig. (3). K-mean analysis of the manually curated dataset. The number of clusters were set to be self-organized and the clusters tend to organize the molecules by the activity over the receptor.

© 2020 Rojas-Rodríguez *et al.*

This is an open access article distributed under the terms of the Creative Commons Attribution 4.0 International Public License (CC-BY 4.0), a copy of which is available at: (https://creativecommons.org/licenses/by/4.0/legalcode). This license permits unrestricted use, distribution, and reproduction in any medium, provided the original author and source are credited.
